# Consistency in the flight and visual orientation distances of habituated chacma baboons after an observed leopard predation. Do flight initiation distance methods always measure perceived predation risk?

**DOI:** 10.1002/ece3.8237

**Published:** 2021-10-17

**Authors:** Andrew T. L. Allan, Annie L. Bailey, Russell A. Hill

**Affiliations:** ^1^ Department of Anthropology Durham University Durham UK; ^2^ Primate and Predator Project Lajuma Research Centre Louis Trichardt South Africa; ^3^ Department of Zoology University of Venda Thohoyandou South Africa

**Keywords:** flight initiation distance, habituation, predation, risk, tolerance

## Abstract

Flight initiation distance (FID) procedures are used to assess the risk perception animals have for threats (e.g., natural predators, hunters), but it is unclear whether these assessments remain meaningful if animals have habituated to certain human stimuli (e.g., researchers, tourists). Our previous work showed that habituated baboons displayed individually distinct and consistent responses to human approaches, a tolerance trait, but it is unknown if the trait is resilient to life‐threatening scenarios. If it were consistent, it would imply FIDs might measure specific human threat perception only and not generalize to other threats such as predators when animals have experienced habituation processes. We used FID procedures to compare baseline responses to the visual orientation distance, FID, and individual tolerance estimates assessed after a leopard predation on an adult male baboon (group member). All variables were consistent despite the predation event, suggesting tolerance to observers was largely unaffected by the predation and FID procedures are unlikely to be generalizable to other threats when habituation has occurred. FID approaches could be an important tool for assessing how humans influence animal behavior across a range of contexts, but careful planning is required to understand the type of stimuli presented.

## Graphical abstract

We used flight initiation distance procedures to compare baseline responses to the visual orientation distance, FID, and individual tolerance estimates assessed after a leopard predation on an adult male baboon (group member). All variables remained consistent, suggesting tolerance to observers was unaffected by the predation event. FIDs may therefore not always be generalisable to other threats once habiutation processes have begun; however, they may still offer an important tool for assessing how humans influence the behaviour of animals in a range of scenarios.

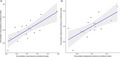

## 1 INTRODUCTION

Flight, fleeing, and escape responses are widely reported antipredator defenses which can directly reduce the chance of an individual being successfully captured (Blumstein, 2003; Blumstein et al., 2003; Cheney & Predation, 1987; Isbell, 1994; Lingle, 2001). Optimal escape theory predicts that the distance at which prey decide to flee from an approaching predator, otherwise known as flight initiation distance (hereafter FID), is governed by a trade‐off between the risk of being predated upon and the benefits of staying to engage in any fitness enhancing activity (Cooper, 2009; Cooper & Frederick, 2007; Cooper et al., 2015; Ydenberg & Dill, 1986). Therefore, increasing risk of predation should correlate with increased FID (Cooper & Frederick, 2007, 2010). Measuring a true FID (in response to an actual predator) is unlikely, and so studies concerning FID and escape behavior have commonly used approaching researchers to measure FID instead (Cooper, 2009; Díaz et al., 2013; Samia et al., 2013; Stankowich & Blumstein, 2005). This is considered valid in most species and scenarios as humans are often considered predators by these animals (Frid & Dill, 2002).

Using human approaches as a surrogate for a predator may be most applicable in areas where humans exert hunting pressure on prey species (Setsaas et al., 2007; Sreekar et al., 2015), although elevated FIDs would also be expected where humans are antagonistic toward animals such as in areas of human–wildlife conflict. However, in these elevated anthropogenic risk scenarios, it is unclear whether FID measures are exclusively measuring human risk perception, or are generalizable to other threats and particularly predators. Using human approaches to measure perceived predation risk appears less justified in areas where interactions with humans are benign, as animals are known to exhibit lower FIDs as a result of habituation processes (Blumstein et al., 2014; Cooper et al., 2015). Reduced FIDs with increasing anthropogenic disturbance have been reported in several birds species (Carrete & Tella, 2011; Díaz et al., 2013; Møller & Tryjanowski, 2014; Morelli et al., 2018, 2019; Samia et al., 2015) and similar effects have been reported in blue‐tailed skinks (*Emoia impar*) (McGowan et al., 2014), fox squirrels (*Sciurus niger*) (Mccleery, 2009), yellow‐bellied marmots (*Marmota flaviventris*) (Li et al., 2011), and mule deer (*Odocoileus hemionus*) (Price et al., 2014). The lower FIDs in these studies suggest diminished risk perception in these animals, but FID measures are still considered to reflect the predictions of economic escape theory (Cooper et al., 2015).

If the reduced FIDs found in areas with higher anthropogenic disturbance are indeed still reflective of an animal's perception of predation risk, then it would suggest that habituation to anthropogenic disturbances can transfer to predators. However, transfer of habituation from humans to predators appears to have only been reported in one instance where urban fox squirrels displayed reduced FIDs in response to human approaches while concurrently exhibiting reduced responses to experimental predator stimuli (Mccleery, 2009); however, the study design has been criticized (Geffroy et al., 2015). In the case of Mccleery, (2009), it could also be argued that increasing anthropogenic disturbance is associated with reduced predator abundance, which offers a selective advantage to tolerant prey animals (Møller, 2012); as such, the reduction in antipredator behaviors may reflect an absence of experience with predators as opposed to habituation transfer (Geffroy et al., 2015). It therefore remains largely unexplored whether diminished risk perception (when quantified by FIDs) is tied specifically to humans. This poses an important question: Do FIDs measure a perception of specific human risks, or do they reflect the risk perception toward other animals (e.g., predators) or risks more generally?

Recently, we explored the visual orientation distance (VOD—the distance at which approached individuals direct their line of vision toward the approaching observer's face) and FID responses of habituated chacma baboons (*Papio ursinus griseipes*) to approaches from observers and found that individuals displayed consistent VODs and FIDs across repeated approaches that were also highly distinct from one another, allowing individual tolerance estimates to be derived (Allan et al., 2020). In addition, the habituated baboons viewed observers as equivalent to a high‐level social threat despite a long history of observations. Here, we build on these analyses and use a naturally occurring predation event by a leopard (*Panthera pardus*) on an adult male baboon from the study group to assess whether habituated animals alter their risk perception of observers after encountering natural predators.

If the VODs and FIDs of the study animals were elevated across postpredation trials, either initially or for several hours postevent, then it would suggest that the habituated baboons altered their risk perception of observers as a result of the predation event. Such a result would indicate that FID methods are appropriate for assessing risk perception in habituated animals during highly threatening scenarios.

Alternatively, if the VODs and FIDs were consistently lower postpredation, it could suggest the animals experienced some form of sensory fatigue. The vigilance decrement described by Dukas and Clark (1995) implies that animals should experience a decline in their cognitive ability to process information effectively through extended periods of time or increasing task difficulty, leading to decreased abilities to detect threats or performance in decision‐making tasks. As the baboons were likely to experience heightened stress levels as a result of the predation, their energetic demands should have also increased, potentially leading to fatigue and lowered risk responses (VOD and FID). Alternatively, reduced VODs and FIDs may also suggest the animals increased their tolerance to observers (or reduced their fear perception of the observers) temporarily as observers are known to displace predators (LaBarge et al., 2020), thus proximity to observers may decrease their likelihood of encountering the predator again, that is, the human‐shield effect (Nowak et al., 2014).

If the predation event had little effect on VOD and FID measures or individual tolerance estimates (of the surviving group members), it would indicate that despite observers being considered equivalent to a high‐level social threat, this threat perception is not altered as a result of the predation. If this prediction is met, it would suggest FID methodology is a robust measure of specific human (i.e., researcher) threat perception only when animals have been habituated, and not generalizable to other threats, which would have implications for research exploring antipredator behaviors using FID methodology in scenarios where habituation processes have taken place.

## 2 METHODS

This research was undertaken under ZA/LP/81996 research permit, with ethical approval from the Animal Welfare Ethical Review Board (AWERB) at Durham University. All data were collected between October and December 2017 on a wild habituated group of Afro‐montane chacma baboons (*Papio ursinus griseipes*) in the western Soutpansberg Mountains, South Africa (central coordinates S29.44031°, E23.02217°). For a detailed study site and group description, see (Allan et al., 2020).

The study group was habituated circa 2005 and was the focus of intermittent research attention until 2014. The study area has experienced long‐term anthropogenic activities (local farming, forestry, and residences) prior to 2005, and so consistent interactions with humans have been ongoing with this population for some time. Since the initial habituation process was completed, several researchers have been able to collect expansive datasets on the study group (Howlett et al., 2015; Raad & Hill, 2019). From 2014, the group received full day (dawn until dusk) follows 3 to 4 days a week, with occasional gaps of up to 5 weeks in duration. The follow schedule was designed to ensure that the study group retained as much of their natural interactions with predators as possible by ensuring that the group had multiple consecutive days without observers who may deter predators or reduce interaction rates (LaBarge et al., 2020; Nowak et al., 2014). During this study, the group contained between 81 and 86 individuals.

The study site was located in a private nature reserve with the majority of the study group's home‐range typically overlapping with the core area of the Lajuma Research Centre which contained numerous camps and residences. Interactions with people living in the area, unfamiliar researchers, and tourists were thus a frequent occurrence. However, the baboons had not engaged in “raiding” residences, threatening humans, or any other potentially negative symptom of habituation prior to the end of this study. The study group were occasionally scared away (chasing, yelling, throwing stones, etc.) from a small plantation by local workers, usually resulting in fleeing responses and sometimes alarm calling. However, the study group appeared adept at recognizing the differences between researchers and these threats and were observed pre‐emptively avoiding them at distances of >200 m. The study group was not hunted during any observation gaps and was the only group habituated for research purposes in the study area with most of the neighboring groups exhibiting strong fear responses to observer sightings, for example, alarm calling and fleeing at distances of >100 m, this was consistent even if observers were following the habituated baboon group.

### 2.1 Nonpredation VOD and FID data

All nonpredation data were taken from our previous study (Allan et al., 2020). To produce an equal sampling effort across the study group, each noninfant individual (*n* = 69) was originally subjected to 12 approaches by each observer (24 in total) varying in familiarity to the study animals. One observer was considered familiar (AA, had followed the group for approximately 3 years), and the second observer was considered unfamiliar (AB, conducted first FID approach on the first day with study group). For this analysis, data generated by AA were excluded; thus, all nonpredation and postpredation approaches were completed solely by the unfamiliar observer, AB. Due to time constraints, only a subset of 16 individuals could be sampled repeatedly (three trials each) postpredation; as such, we only utilized data from the same 16 individuals for our nonpredation data. These nonpredation trials were used as a baseline for comparison to explore whether the predation event could alter the baboon's typical VOD and FID responses and ultimately tolerance to human observers.

### 2.2 Predation event

Baboons form a significant component of leopard diets in the study region (Chase Grey et al., 2017; Williams et al., 2018). Group‐wide alarm calls spread across the study group on November 2, 2017, at 11:09. Shortly after, we discovered the body of an adult male baboon (group member) that had been predated by a leopard on the periphery of the group. The predation event was not directly observed, but inspection of the body indicated the baboon had received a kill bite to the nape of the neck. The baboon was completely motionless by the time of our arrival (approximately half the group was already there), and within minutes, the remaining group members (86 at the time) had grouped around the dead baboon. At this stage, several individuals were directing alarm barks into the bush nearby where the leopard likely retreated, movement could be heard in the bush at this stage, and both AA and AB heard the short “sawing” vocalization typically made by leopards, although the sound was brief and masked by the intensity of the baboon vocalizations. Most group members inspected the body before moving away and looking toward a bush that the leopard may have retreated into. By 11:42, the group had begun dispersing and reengaging in foraging behaviors, except for two adult males who remained with the body. We marked the end of the alarm state in the group as ending at 11:48. At approximately 12:14, the remaining males had rejoined the rest of the group and all individuals continued foraging and moving through a typical part of their range for the afternoon. Camera traps set around the body confirmed the leopard returned a couple of hours later to drag the baboon away.

### 2.3 Postpredation study design

At 11:58, we began a series FID approach trials on a subset of 16 individual group members that evenly represented a number of age–sex classes (six adult females, two adolescent females, two juveniles females, one adult male, one adolescent male, four juvenile males). Age–sex class ratios were briefly approximated after the predation event, and focal animals selected from these age–sex classes pseudorandomly. All individuals were assigned random numbers in an excel spreadsheet (mobile device) and the individuals with the highest values for each age–sex class selected from the list. Individuals retained their random numbers and the first individual in the top five on the list was approached when encountered. Once a trial was completed, this animal was assigned a new random number and reintegrated at the end of the list. As such, some individuals received their 2nd and 3rd trials before others had received their 1st and 2nd trials, respectively. Each individual was approached three times during the remainder of the day (48 total trials). These postpredation approaches were unique to this study and not used in the previous analysis. The first trial was completed 10 min after the alarm state had ended, and the last trial was 367 min after the alarm had ended, while the average time since the alarm ended was 186 min.

### 2.4 FID approach procedures

The effect of start distance on FIDs has received a great deal of attention in FID research and is one of the strongest and most consistently reported effects (Dumont et al., 2012). It has been recommended that the start distance chosen by observers be systematically varied to achieve a true understanding of the dynamics of flight responses (Blumstein et al., 2015). As such, we attempted to distribute our start distances evenly from close (approximately 3 m) to distant (8 m and beyond) for each individual. This range of distances was chosen as they reflected typical observation distances. Most individuals received an even distribution of approach distances for nonpredation data (Allan et al., 2020); however, certain intolerant individuals did not permit close start distances. For postpredation data, some individuals did not receive a wide distribution of start distances (see dataset) due to the lower number of trials and time constraints limiting the opportunities to complete longer approaches on all subjects, that is, the further the intended start distance, the more likely obstructions and other baboons would be between the observer and the focal. As such, it was more challenging to complete these approaches.

When a focal animal was encountered in a stationary behavior, the observer selected a start position for the approach according to the focal animal's prior distribution of start distances. This start position had to be within a 90° field of view of the front of the focal animal's head (45° either side of center), that is, the animal's face had to be broadly facing forward toward the start position. If approaches were completed outside of the animal's likely field of vision, then it would be challenging for the animal to detect the observers visually, forcing them to rely on other stimuli for detection. Before commencing each approach, the observer waited for at least 10 s at the start position before beginning an approach and would only commence if there was no response from the focal animal to our presence within this time frame. We abandoned a trial if another baboon sat between the observer and the focal animal prior to the start of the approach, or if the focal animal turned their head such that we could no longer approach within their field of vision, or the focal was already looking toward either of the observers. Whenever an approach was aborted, another focal animal was selected instead.

We did not vary approach speed systematically and instead attempted to achieve consistent walking pace across all approaches as this mimicked typical observer behavior while observing the study animals. When approaching focal animals, the observer was required to focus their gaze on the animal's forehead to maintain the same speed and posture throughout the approach (Runyan & Blumstein, 2004), avoid tripping and falling, and to allow the observer to easily identify each behavioral assessment (i.e., visual orientation, neighbor movement, and flight). Direct eye contact was avoided as this can startle baboons and may mimic their typical dominance behaviors. We did not attempt any approaches when the animals were close to large obstructions (e.g., building, rocks, large trees) or cliff edges, as these limit flight options. We made no approaches if there were obstructions between the observer and the focal animal to ensure posture and approach speed remained constant and to ensure the animal was not alerted prematurely by the sound of observers brushing past vegetation or obstructions.

When ready to start an approach, the observer (AB) dropped a small painted stone (approx. 2 cm in diameter) behind their feet to mark the start distance and dropped further stones to measure VOD and FID. VOD was defined as the focal animal directing their line of vision toward the face of the approaching observer, while FID required the animal to move away from its original position as a direct result of the approach. In all approaches, the observer walked directly to the focal animal's start position without pausing at any point. In order for an FID observation to be valid, it required the animal to visually orient toward the observer before displacing; otherwise, it would have been unclear if the animal's movement was a direct response to the approach; however, we had no trials where displacement was not proceeded by visual orientation. We also had no instances of nonfocal animals crossing our path as we made our approaches, but approaches would have been abandoned in these situations.

A second observer (AA) was always present further away than the approaching observer to ensure stones landed in accurate locations and to assess the range of contextual variables. We observed no reactions to stones landing on the ground, this was either because the observer's footsteps masked the noise of stones hitting the ground, or the sound was an insufficient stimulus to warrant visual orientation or displacement in any trial; however, if we had observed this, we would have abandoned the trial. Distances between markers and the start position of the focal animal were then measured using a calibrated laser range finder (Leica DISTO DXT) and recorded on an electronic device (Samsung Galaxy J5, Samsung Town, Seoul, Republic of Korea), using a personalized application built with the software CyberTracker v3.466 (CyberTracker Conservation, Bellville, South Africa; http://www.cybertracker.org). After the approach was successfully completed, we noted the behavioral response of the focal animal (behaviors listed in Allan et al. (2020)).

### 2.5 Contextual variables

Baboons are able to change between a range of behaviors rapidly; therefore, we elected not to restrict approaches to certain behaviors. Instead, we used an instantaneous point sampling method to record behavioral, social, and environmental variables at the instant we commenced an approach. We recorded the following factors: If the animal was performing engaged (foraging, autogrooming, and giving grooming) or nonengaged (resting, chewing, receiving grooming) behaviors, looking or not looking, habitat type (open/closed), and number of neighbors within 5 m. We chose 5 m as the proximity buffer for recording the number of neighboring conspecifics as this was a frequently used measurement in other research conducted by AA (which had been validated previously) and reflected a compromise between collecting the most amount of information in high‐visibility locations and minimizing sampling issues in low‐visibility locations. Habitat descriptions are detailed in Allan et al. (2020) but briefly, closed habitats were forest, woodland, or bushland habitats characterized by dense woody vegetation, while open habitats were largely devoid of similar obstructions and had much higher visibility, including grassland, roads, trails, camps, and rocky areas.

Looking was defined as the focal animals’ eyes being open and their line of vision extending beyond their hands and the substrate, animal, or object their hands were in contact with (Allan & Hill, 2018, 2021) for discussion). The premise of this definition is to ensure as much information about the baboons general looking behaviors are recorded, assuming that multiple information acquisition pathways are compatible. For example, an individual looking toward a distant group member likely has more chance of detecting an approaching threat than an individual engaged in a complex foraging task. Our previous results supported that individuals looking at the initiation of our approaches visually oriented the quickest, and individuals not looking due to being engaged in other tasks (e.g., foraging or grooming) had typically longer VODs (Allan et al., 2020). We also noted the trial number the animal had received so far in the study and during the observation day. Approaches were made across the full range of habitats the study group utilized (Allan et al., 2020 for descriptions); we did not manage to sample each individual evenly across each habitat type.

### 2.6 Statistical analysis

We broke the analysis into two separate approaches. Firstly, we analyzed nonpredation data (*n* = 192 trials) and postpredation data (*n* = 48 trials) separately. In the second approach, we combined all observations into a single dataset. In each approach we created separate models for each response variable—visual orientation distance (VOD) and flight initiation distance (FID)—leading to 3 models for each variable (and six total): nonpredation data, postpredation data, and combined data. We used Bayesian mixed model analysis to explore a number of potential factors that could influence the VOD and FID of individual baboons. The most informative predictors of Allan et al. (2020) were used in all models; engaged/not engaged behaviors were used only in FID models, while compatibility (i.e., engaged, not engaged not looking, or looking) was only used in VOD models. Habitat type and number of neighbors were included in all models. We did not use animal height from our previous study as “above ground” was not well observed in postpredation trials. Observer identity was not used as trials were only completed by one observer (AB) in this study. The variables of external encounters, neighbor flee first were also removed as they previously offered little predictive power (Allan et al., 2020) and we did not want to overparameterize models on smaller datasets.

Time period was included as a covariate in all models as postpredation data occurred during the afternoon, and VOD and FID could vary across the day. Time periods were seasonally adjusted to reflect 25% of current day length; all nonpredation data were sampled evenly within each time period at an individual level. For models utilizing the nonpredation data, individual trial number was included as a numeric variable (as described in Allan et al., 2020). We did not include any variables for time since predation or group trial number post predation as these both accumulated across the group after the event and would therefore be challenging to incorporate into a design focusing on changes at the individual‐level. Instead, trial number postpredation was included as a factor variable so that the mean conditional effects of each wave of trials could be easily visualized. In nonpredation and postpredation models, trial number was also included as a random slope over individual identity to allow the rate at which individuals habituated/sensitized to the approach stimulus to vary between individuals.

Models utilizing the combined nonpredation and postpredation dataset were the same except we also included trial type (nonpredation or postpredation) as a population‐level effect and as a random slope over individual identity, thus allowing the individual responses to the predation stimulus to be modeled. Using total individual trial number (1–12 for non‐predation data, 1–3 for post‐predation data) would have led to misleading results, as such we instead included individual trial number per day as a population‐level effect and as random slope over individual identity for both models using the combined datasets. Date was also included as a group‐level factor as each individual (*n* = 16) was also sampled in the morning prior to the predation event, allowing us to control for any variance that could be explained by observation date. For all VOD models, we included the difference between the start distance and VOD (visual orientation distance delay) as a fixed effect and a random slope, while FID models included the difference between VOD and FID (visual orientation distance interval) as a fixed effect and a random slope; in both cases, the random slope varied over individual identity. This is a recommended approach to control for the constrained envelope issues found in typical FID analyses as both variable are independent of start distance (Allan et al., 2020; Bonnot et al., 2017).

All models were fit using the brms package (Bürkner, 2017). For models using nonpredation data and the combined datasets, a lognormal response distribution was defined, while a Gamma distribution was defined with log link functions for postpredation models. In all cases, Student‐t default priors (*df* = 3, *M* = 0, scale parameter = 10) were assigned to all components in the brms models; however, the standard deviations of group‐level effects were constrained to be positive and therefore assigned a half Student‐t prior. Non‐predation models were run for four Hamiltonian Markov chains for 10,000 iterations, both set higher than default settings to aid fitting a relatively small sample size and allowing algorithms to converge efficiently (Bürkner, 2017). Warm‐up iterations were set to 4000 and adapt_delta to 0.9, both greater than default to aid in producing robust posterior samples from smaller datasets (Bürkner, 2017; McElreath, 2019). For combination models and postpredation models, the number of chains was increased to 6, warm‐up was increased to 6000, and adapt_delta to 0.95 for combination models and 0.999 for postpredation models.

The Gelman–Rubin convergence diagnostic (Rhat) was used to assess Markov chain Monte Carlo convergences. This is achieved by comparing the estimated within‐ and between‐chain variances of all factors within the models. Rhat was equal to 1.00 in all cases suggesting the standard deviation of points formed around the lognormal and gamma functions were minimal. For each model, we inspected the bulk and tail effective sample sizes (ESS); bulk ESS estimates the sampling efficiency for the mean of each distribution, while tail ESS computes the minimum effective sample sizes of the 5% and 95% quantiles. In all cases, the bulk ESS was greater than 100 times the number of chains, and the tail ESS were similarly high, indicating the posterior distributions were well estimated and there were no issues relating to different scales of chains or slow mixing of within the tails of the distributions (Bürkner, 2017). We extracted the conditional modes (known as best linear unbiased predictors elsewhere (Carter et al., 2012) of each individual baboon from each nonpredation and postpredation model, yielding individual visual and displacement tolerance estimates (Allan et al., 2020) for non‐ and postpredation data.

## 3 RESULTS

**FIGURE 1:**
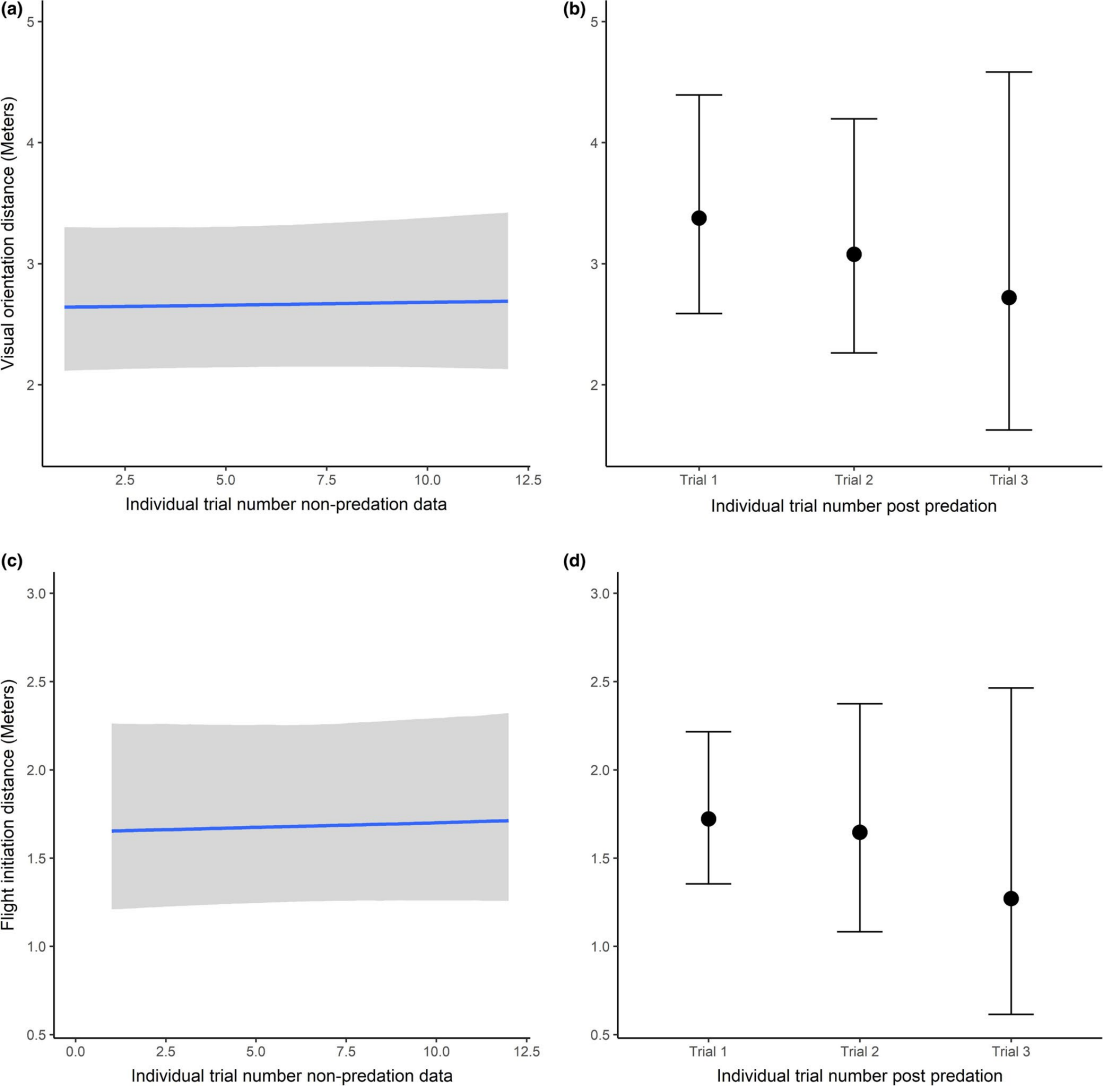
Mean conditional effects for visual orientation distance and flight initiation distance: (a) the relationship between VOD and trial number for nonpredation data; (b) the mean conditional effect of VOD for each trial after the predation event; (c) the relationship between FID and trial number for non‐predation data; (d) the mean conditional effect of FID for each trial number after the predation event. The shaded areas in (a) and (c) and the tails in (b) and (d) display the relevant credible intervals (2.5 and 97.5 percent quantiles)

Visual orientation distance was consistent through successive trials in the nonpredation dataset (Figure 1). Although there was a slight increase in VOD (quicker detection) for trial number 1 after the predation event, the mean conditional effect for trails 2 and 3 postpredation fell within the upper and lower 95% credible intervals for the nonpredation data. While VOD was consistent through time periods 3 and 4 after the predation event (see Appendix S1: Fig A1), the mean conditional effect for time period 4 postpredation was marginally higher than the upper credible interval for time period 4 in the nonpredation data. Full summary results for each model are shown in Appendix S1: Tables A1 and A2.

Flight initiation distance was consistent through successive trials in the nonpredation dataset (Figure 1), with a mean conditional effect of 1.68 (1.25, 2.27). The mean conditional effect postpredation was very similar: Trial 1 was 1.72 (1.35, 2.22), trial 2 was 1.65 (1.08, 2.37), and trial 3 was 1.27 (0.61, 2.46). Although the mean conditional effect for trial number 3 was slightly lower, the mean fell within the credible intervals of the nonpredation trials. FID was also consistent through time periods 3 and 4 after the predation event (see Appendix S1: Fig A2), with the conditional means of both time periods for postpredation models falling within the credible intervals of the respective time periods in the nonpredation models. Full summary results for each model are shown in Appendix S1: Tables A3 and A4.

When the nonpredation and postpredation datasets were combined, we also found no evidence that VOD or FID were influenced by the predation at the population‐level, with both estimates close to zero and 95% credible intervals (CI) overlapping zero for the postpredation dataset (see trial type (postpredation): Tables 1 and 2). For both response variables, the results produced for engaged, compatibility, habitat, and number of neighbors matched the findings previously reported in Allan et al. (2020).

**TABLE 1:** VOD parameter estimates for the model describing the relationship between VOD and the predictor variables

	Estimate	Est. Error	l−95% CI	u−95% CI	Rhat	Bulk_ESS	Tail_ESS
Population‐level effects							
Intercept	1.2	0.12	0.96	1.43	1.00	9567	14,205
Visual orientation distance delay (VODD)	−0.06	0.03	−0.13	0	1.00	10,359	13,976
Compatibility (Looking)	0.08	0.05	−0.01	0.17	1.00	19,554	19,133
Compatibility (Not looking not engaged)	0.03	0.07	−0.1	0.16	1.00	20,977	18,109
Habitat (Open)	0.18	0.05	0.09	0.27	1.00	14,758	16,825
Number of neighbors in 5 m	−0.05	0.02	−0.08	−0.02	1.00	19,391	18,317
Time period (2)	0.01	0.06	−0.11	0.12	1.00	17,354	18,025
Time period (3)	0.11	0.06	−0.01	0.23	1.00	13,888	17,649
Time period (4)	−0.01	0.07	−0.15	0.12	1.00	11,829	16,319
Trial type (Post predation)	0.04	0.12	−0.19	0.27	1.00	16,567	16,600
Individual trial number per day	−0.02	0.05	−0.11	0.07	1.00	14,857	15,683
Family specific parameters							
Sigma	0.25	0.02	0.23	0.29	1.00	8207	14,761
Group‐level effects							
Date (28 levels)							
sd(Intercept)	0.18	0.05	0.09	0.29	1.00	4503	5289
Individual identity (16 levels)							
sd(Intercept)	0.32	0.09	0.18	0.52	1.00	9409	14,780
sd(VODD)	0.09	0.03	0.03	0.17	1.00	5390	5741
sd(Trial type—postpredation)	0.15	0.1	0.01	0.39	1.00	5071	9225
sd(TrialNoDay)	0.07	0.04	0	0.17	1.00	4190	7037
cor(Intercept,VODD)	0.02	0.33	−0.57	0.68	1.00	7770	11,535
cor(Intercept, Trial type—postpredation)	0.09	0.39	−0.69	0.79	1.00	18,206	16,304
cor(VODD, Trial type—postpredation)	0.07	0.42	−0.75	0.81	1.00	13,657	16,506
cor(Intercept, TrialNoDay)	−0.1	0.39	−0.78	0.69	1.00	14,104	15,409
cor(VODD, TrialNoDay)	−0.24	0.4	−0.88	0.62	1.00	11,742	14,945
cor(Trial type—postpredation, TrialNoDay)	−0.25	0.45	−0.91	0.7	1.00	7445	14,404

Abbreviation: CI, credible interval.

**TABLE 2:** FID parameter estimates for the model describing the relationship between VOD and the predictor variables

	Estimate	Est. Error	l−95% CI	u−95% CI	Rhat	Bulk_ESS	Tail_ESS
Population‐level effects							
Intercept	0.75	0.16	0.44	1.08	1.00	6985	11,382
Visual orientation distance index (VODI)	−0.1	0.04	−0.18	−0.01	1.00	19,771	16,016
Engaged (Not engaged)	0.12	0.05	0.02	0.22	1.00	26,547	18,721
Habitat (Open)	0.15	0.06	0.03	0.26	1.00	19,000	18,600
Number of neighbors in 5 m	−0.07	0.02	−0.11	−0.03	1.00	26,317	18,039
Time period (2)	0.01	0.07	−0.13	0.16	1.00	22,979	18,986
Time period (3)	0.13	0.08	−0.02	0.28	1.00	18,205	18,364
Time period (4)	−0.03	0.08	−0.19	0.13	1.00	18,361	18,773
Trial type (postpredation)	0.14	0.15	−0.16	0.43	1.00	16,137	16,164
Individual trial number per day	−0.05	0.06	−0.16	0.06	1.00	19,150	17,507
Family specific parameters							
Sigma	0.33	0.02	0.29	0.37	1.00	11,566	15,544
Group‐level effects							
Date (28 levels)							
sd(Intercept)	0.16	0.06	0.06	0.28	1.00	5723	5709
Individual identity (16 levels)							
sd(Intercept)	0.51	0.12	0.33	0.8	1.00	9910	13,458
sd(VODI)	0.09	0.05	0.01	0.21	1.00	6022	9580
sd(Trial type ‐ post predation)	0.17	0.12	0.01	0.44	1.00	8606	12,271
sd(TrialNoDay)	0.09	0.05	0.01	0.21	1.00	5471	9634
cor(Intercept,VODI)	0.11	0.37	−0.61	0.8	1.00	22,550	15,825
cor(Intercept, Trial type—postpredation)	−0.23	0.4	−0.86	0.63	1.00	22,709	16,218
cor(VODI, Trial typepostpredation)	−0.04	0.44	−0.82	0.79	1.00	19,082	18,381
cor(Intercept, TrialNoDay)	−0.33	0.36	−0.87	0.53	1.00	18,947	15,477
cor(VODI, TrialNoDay)	−0.1	0.42	−0.83	0.73	1.00	13,522	15,406
cor(Trial type—postpredation, TrialNoDay)	−0.18	0.45	−0.88	0.73	1.00	9922	16,938

Abbreviation: CI, credible interval.

**FIGURE 2:**
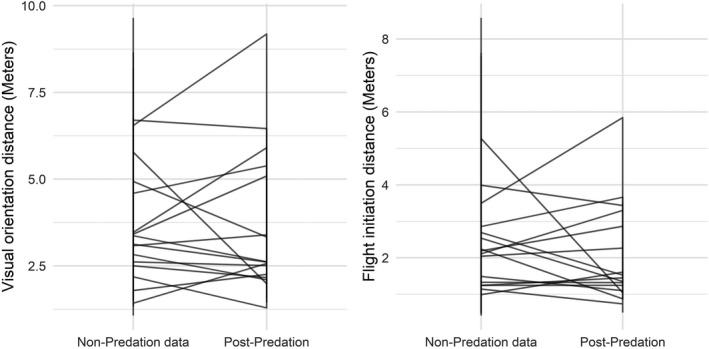
Line graphs representing the predicted individual‐level means for VOD and FID for nonpredation and postpredation data

The group‐level effects highlight that there was no evidence that the slopes varied according to trial type (i.e., cor(Intercept, Trial type—postpredation), for example, there was not a consistent trend for individuals with typically higher or lower VODs during nonpredation trials to produce longer or shorter VODs postpredation. Although a small number of individuals exhibited positive or negative slopes across the nonpredation and postpredation datasets (see Figure 2), the differences were minimal for most individuals, further supporting that the predation event had little influence on the typical VODs and FIDs of the individuals used in this study.

**FIGURE 3:**
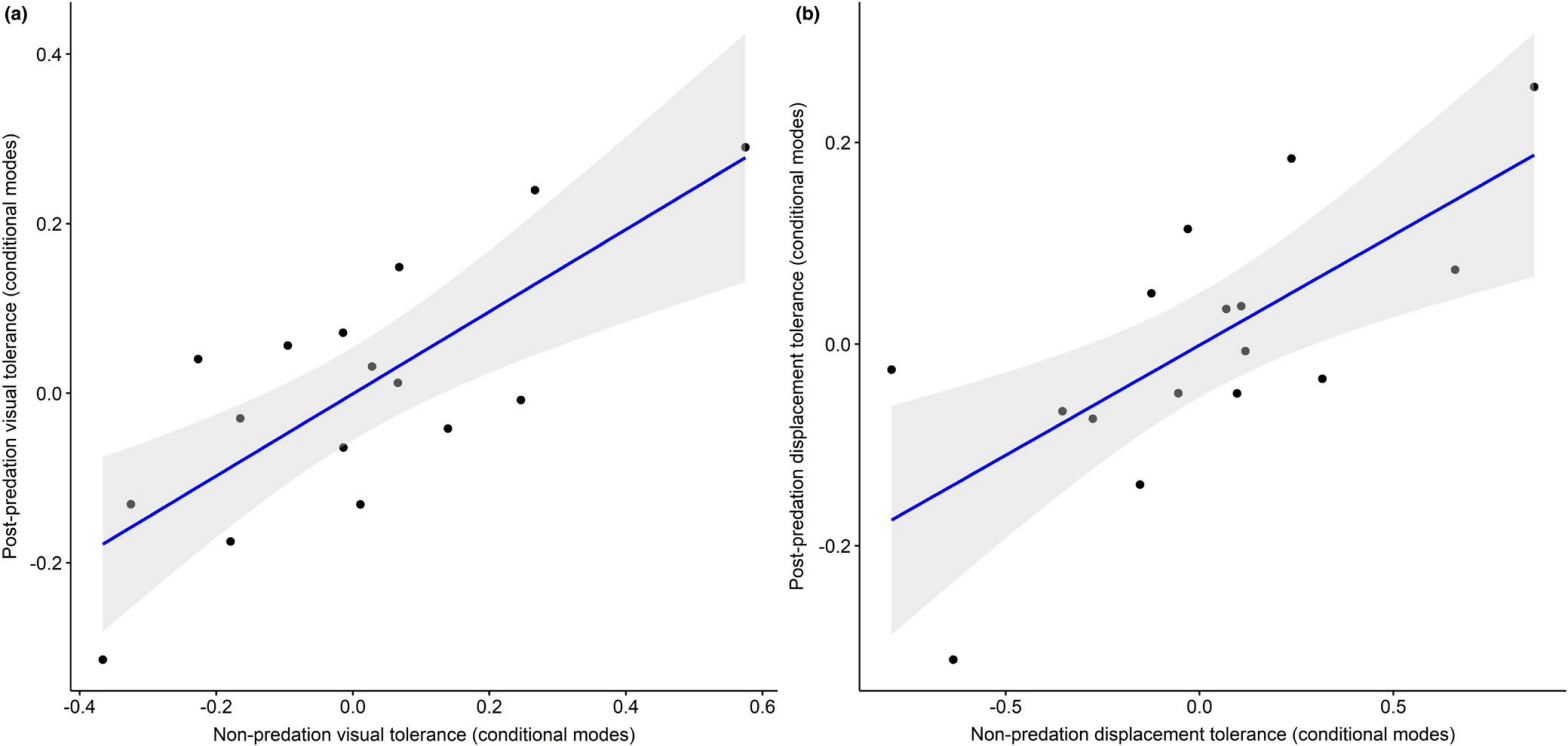
Nonpredation and postpredation correlations for conditional modes of each tolerance measure: (a) correlation between individual‐level visual tolerance estimates (derived from VOD measures); (b) correlation between individual‐level displacement tolerance estimates (derived from FID measures). Lower/negative estimates indicate greater tolerance. Conditional modes for nonpredation data were calculated from the nonpredation models, and postpredation conditional modes from the postpredation models

Finally, we also found good correlation between individual tolerance estimates (i.e., conditional modes) between nonpredation data and postpredation data (Figure 3; visual tolerance correlation: (*r*(14) = 0.76, *p* = .001); displacement tolerance correlation: (*r*(14) = 0.703, *p* = .002)), highlighting that tolerance was consistent despite the predation event. We also found that the conditional modes generated from the postpredation trials were consistent with the conditional modes reported in Allan et al. (2020) (visual tolerance correlation: (*r*(14) = 0.80, *p* = .001); displacement tolerance correlation: (*r*(14) = 0.68, *p* < .001)) despite the previous study utilizing an additional 12 approaches (for each individual) from another observer differing in physical characteristics and familiarity with the study animals.

## 4 DISCUSSION

We examined the visual orientation and flight responses of habituated chacma baboons to approaches made by observers, comparing responses from after a leopard predation event to data collected during less threatening and stressful scenarios. The predation event had little effect on either variable or individual tolerance estimates. However, some minor effects were discernible—the predation event seemed to make individuals slightly nervous (quicker visual orientation) for a short period—but this seemed to return to a normal level during subsequent trials. Furthermore, even though visual orientation was initially quicker, FIDs were largely unchanged following the predation, suggesting the baboons still viewed observers as a high‐level social threat and that this was unaltered despite being slightly more primed to detect threats shortly after the predation event. Although derived from a single predation event, these results suggest that human approaches (using FID methodology) measure a very specific threat perception relating to humans when animals have experienced habituation processes and most of their human encounters are benign. As such, FIDs may not be generalizable to other forms of risk (i.e., predation) once habituation processes are underway.

Due to the intensity of the predation event, the subsequent alarm state in the study group, and the time needed to formulate an effective study design, our first approach took place 49 min after the predation event—10 min after we considered the alarm state to have mostly subsided. It is possible that if approaches were commenced immediately after the predation event, then our results may have been different, although the ethical and safety implications of undertaking such approaches made it unfeasible. Nevertheless, Engh et al. (2006) reported that female chacma baboons who lost a relative had increased fecal glucocorticoid levels in the four weeks that followed predation events relative to baseline levels, before returning to baseline levels in the subsequent month. Although the observed increase could be attributed to a loss of a social partner, these females adapted to the loss by increasing their grooming time and diversifying the number of grooming partners. As such, it was interpreted that the physiological stress endured due to a loss of a relative was likely mitigated by the behavioral adjustments, suggesting the increase in glucocorticoid levels was partly due to lingering effects of the predation directly. In gray‐cheeked mangabeys (*Lophocebus albigena*), it was found that males generally exhibited increased cortisol levels the day after encounters with crowned eagles (Arlet & Isbell, 2009), while the stress response of captive chimpanzees to anesthesia resulted in increased fecal cortisol concentrations for two days poststress stimulus (Whitten et al., 1998). It seems likely, therefore, that our study animals experienced heightened stress levels for the duration of our postpredation approaches and so the lack of changes in VOD and FID is unlikely to be because trials began too long after the predation event.

Some individuals may have found the event more stressful than others due to witnessing the event directly (or at least detecting the leopard), while others may have had stronger social connections with the predated animal (Engh et al., 2006). Although these factors could not be explored formally in this study, we did observe that several individuals who were present at the predation site before our arrival appeared incredibly agitated for some time after the event (alarm barks continued after leaving the predation site). Despite this, individual VODs and FIDs remained relatively consistent, while individual tolerance estimates (assessed using conditional modes) were also relatively consistent for most individuals.

Despite their tolerance of observers and finding no detectable differences in responses to familiar or unfamiliar observers previously (Allan et al., 2020), the habituated baboons consistently fled at the site of workers from a local farm (often without a behavioral driver such as chasing or throwing stones by the workers). This suggests that even in relatively stable settings where human–primate interactions are normally benign, that baboons still distinguish between classes of humans and their potential risks. In addition, the habituated group still exhibited intense alarm and agonistic responses (e.g., chase and attack) to foreign individuals/groups of baboons and leopards, rock pythons, and crowned eagles (*AA*, *personal observations*), strongly suggesting there was no evidence of habituation transfer in this group. Individuals in hunted and nonhunted primate populations can apparently distinguish between human groups (e.g., hunters, gatherers, researchers) and display diminished responses to lower threats, such as researchers (Papworth et al., 2013). This implies that FID researchers would need to carefully mimic the appearance and behavior of hunters to generate true indications of hunting pressure, at least in wild primates, although other species have been shown to discriminate between human stimuli, for example, snorkelers vs spearfishers in fishes (Sbragaglia et al., 2018) and familiar vs unfamiliar human stimuli in Asian elephants (*Elephas maximus*) (Polla et al., 2018). Some caution may need to be applied however, as FID research also indicates strong habituation effects to FID approaches (Petelle et al., 2013); thus, in species that struggle to differentiate between human stimuli, habituation to FID approaches could also enhance hunting success.

Thought must also be given to landscape‐level habituation, such as proximity to camps, trails, recreational areas, and how it can interact with individual‐level habituation to FID methodology. For example Petelle et al. (2013) reported reduced FIDs in *M*. *flaviventris* colonies that typically received greater anthropogenic disturbance, while also reporting that individual FIDs decreased with increasing trial number for both yearlings and adults across all colonies; indicating two distinct habituation processes had influenced the FIDs of study animals. Results such as these highlight numerous dimensions to habituation/sensitization processes that need to be measured to capture the true impact of approach methodology and understand the fear perception individual animals have toward multiple human stimuli (Allan et al., 2020).

Outside of direct observations on habituated animals, anthropogenic disturbance is likely to vary in type (e.g., hunter, researcher, tourists), intensity (i.e., consistent, sporadic, rare), and outcome (i.e., benign, life‐threatening), all of which could vary temporally at the individual‐level. As a result, habituation/sensitization process are likely to be ongoing in most wild animals (Blumstein, 2016). Investigating individual consistency through time should be an important avenue for future research to explore; however, care must be taken to ensure approaches do not engineer phenotypes that are more vulnerable to human–wildlife conflict and hunting.

Although we present data from a single group and after only one predation event, the 192 nonpredation trials (12 approaches per individual) are beyond the norm in FID research (in terms of individual sampling effort), while our 48 postpredation observations (3 approaches per individual) is similar to sample sizes in the small number of studies that have achieved multiple approaches on known individuals (Carrete & Tella, 2010, 2013; Runyan & Blumstein, 2004). We highlight that our previous study (Allan et al., 2020) demonstrated the individual consistency in VOD and FID measurements for this study group of baboons (69 individuals received 24 trials each, *n* = 1656 total trials) across a range of environmental (e.g., habitat type), social (e.g., number of neighbors), and methodological scenarios (e.g., observer familiarity, trial number), across multiple years (Allan et al.). While future research following other opportunistic predation events would be beneficial, the broader research surrounding our current results adds confidence to our findings.

Typically, FID research has explored anthropogenic disturbance and risk hypotheses on dichotomous landscape‐level axis, such as urban vs nonurban areas, for example, (Uchida et al., 2016). Elsewhere, inferences about FIDs have been made based on observations from inside vs outside protected areas, for example, (Gotanda et al., 2009), or across areas varying in predation pressure, for example, (Januchowski‐Hartley et al., 2011). Experimental designs have also been used to monitor the long‐term FID responses of different groups of animals released into exclosures with and without predators (West et al., 2018) and to compare FIDs of animals across anthropogenic disturbance gradients in response to typical human stimuli and to novel predators (Rodriguez‐Prieto et al., 2009). Our results offer a preliminary perspective on a different axis, exploring how individual VODs and FIDs are modified immediately after encountering a natural predator. Although our study utilized a naturally occurring event, a similar approach could be used to track individual responses to experimental predator encounters, habitat modifications, or changes in anthropogenic disturbances and would be an effective way of tackling outstanding FID questions. For example, assessing individual FIDs in rural settings prior to urbanization could reveal whether intolerant individuals habituate to anthropogenic disturbance or whether urbanization selects for more tolerant or bolder phenotypes (Geffroy et al., 2015).

In the nonpredation dataset used in this study, the least tolerant animals had an average VOD of 6.17 m and an average FID of 5.01 m. It is clear, therefore, that our study group was well habituated. Even so, some individuals were still less tolerant than examples reported elsewhere in FID research. For example, the lowest FID distance for burrowing owls was 4 m in Carrete and Tella (2010) and 3.5 m in Carrete and Tella (2013), average FIDs of 1 m were reported in some populations of European birds (Díaz et al., 2013), several bird species had average FIDs of <5 m in eastern Australia and Tasmania (Blumstein, 2003), while some agama lizards (*Agama planiceps*) (Carter et al., 2012) and coral reef fishes (Januchowski‐Hartley et al., 2011) allowed approaches to within half a meter. As such, the habituation level of our study baboons is unlikely to substantially beyond the level exhibited by animals used in prior FID studies. This suggests our results should be applicable to animals that have begun habituation processes as a result of urbanization, tourism, or any other consistent but benign exposure to humans. Future FID research should attempt to integrate repeated approaches on a diverse range of individually identifiable phenotypes to ensure that assessments of fear perception are not biased by oversampling individuals within particular tolerances toward humans, allowing greater insights into whether FID approaches truly capture general risk perception in all scenarios.

In conclusion, our results suggest habituated chacma baboons display individual tolerance levels (toward observers) that are consistent even after predation events. Most FID research has so far worked on the assumption that humans are considered equivalent to predators (Frid & Dill, 2002), but, in an ever‐urbanizing world, this may only rarely be the case. Future FID studies will need to take care when assuming their approaches are measuring other types of perceived risk, as our results suggest FID methods may only measure very specific types of human‐risk when habituation has taken place. Given the variability in individual tolerances (Allan et al., 2020), future work utilizing human approaches will need to incorporate an individual‐level focus to truly ascertain the anthropogenic impact of the study methodology and how it interacts with other forms of ongoing anthropogenic disturbance. In such scenarios, FIDs may not represent all forms of risk perception, but carefully designed studies could improve our understanding of the impact humans have on animals in a range of scenarios.

## CONFLICT OF INTEREST

We declare no conflicting interests.

## AUTHOR CONTRIBUTION

**Andrew T. L. Allan:** Conceptualization (equal); Data curation (supporting); Formal analysis (lead); Funding acquisition (equal); Investigation (equal); Methodology (equal); Project administration (lead); Resources (lead); Supervision (lead); Validation (equal); Visualization (equal); Writing‐original draft (lead); Writing‐review & editing (equal). **Annie L. Bailey:** Conceptualization (equal); Data curation (lead); Formal analysis (supporting); Investigation (equal); Methodology (equal); Validation (equal); Visualization (equal); Writing‐original draft (supporting); Writing‐review & editing (equal). **Russell A. Hill:** Formal analysis (supporting); Funding acquisition (equal); Investigation (supporting); Methodology (supporting); Resources (equal); Supervision (equal); Writing‐review & editing (equal).

## Supporting information

Appendix S1

## Data Availability

All necessary data required to produce all analyses reported in this article are available on dryad (https://doi.org/10.5061/dryad.83bk3j9s8).
