# Supplementary material for: Consistency in the flight and visual orientation distances of habituated chacma baboons after an observed leopard predation. Do flight initiation distance methods always measure perceived predation risk?
Source: Ecol Evol. 2021 Oct 17;11(21):15404–16. doi: 10.1002/ece3.8237 (PMC8571578; doi:10.1002/ece3.8237)
Supplement: Supplementary file 1 — Appendix S1 [file ECE3-11-15404-s001.docx]

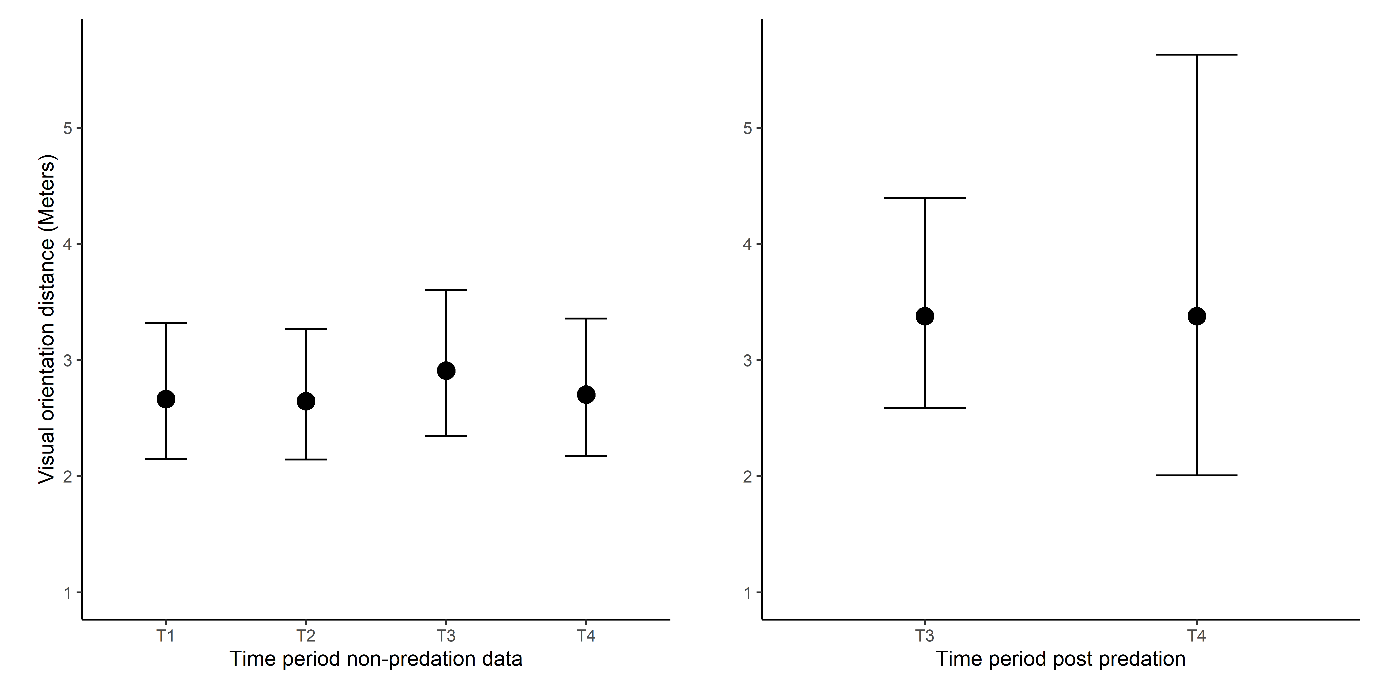
**Appendix**

**Fig A1.** Mean conditional effects for visual orientation distance and time period, panel (a) represents non-predation data, whilst panel (b) represents post-predation data.


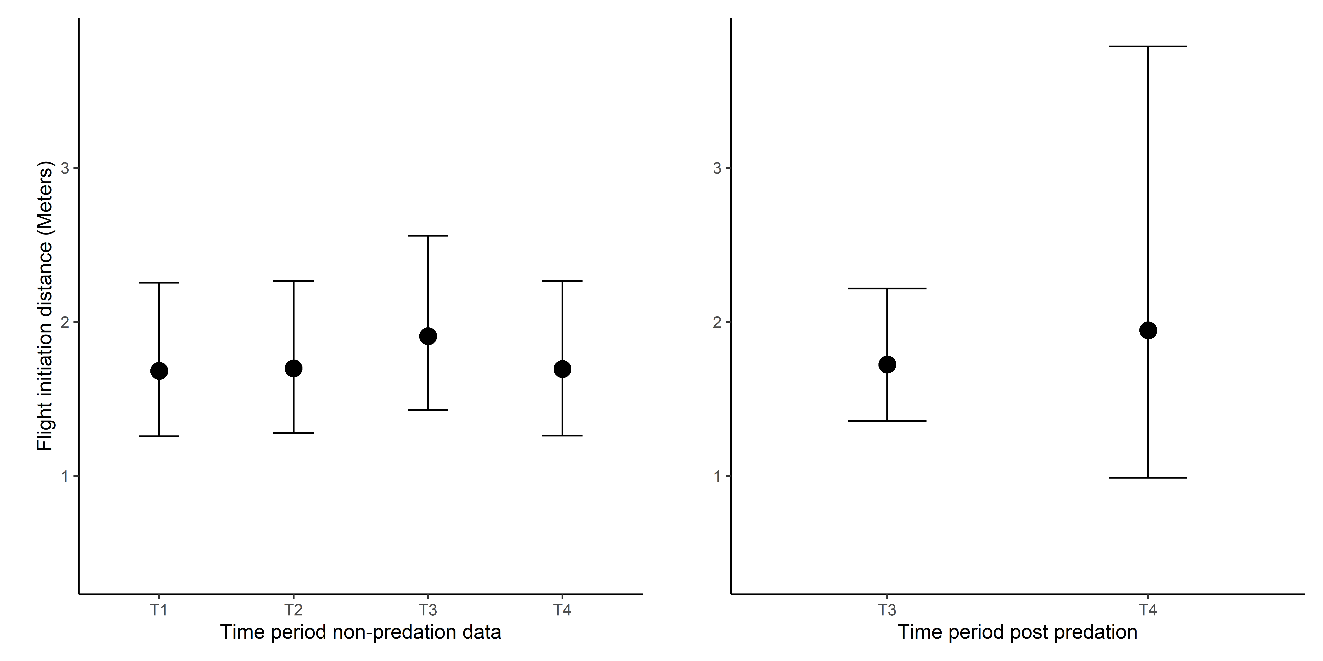
The mean VOD conditional effect (see fig A1) of time periods 3 and 4 for non-predation data were 2.89 (2.34,3.59) and 2.69 (2.16,3.33). Whilst the respective information for post-predation data was 3.38 (2.59,4.39) and 3.38 (2.01,5.63), representing a mean conditional effect difference of 49cm and 69cm (between non-predation data and post-predation data) for time period 3 and 4 respectively.

**Fig A2.** Mean conditional effects for flight initiation distance and time period, panel (a) represents non-predation data, whilst panel (b) represents post-predation data.

The mean FID conditional effect (see fig A2) of time periods 3 and 4 for non-predation data were 1.91 (1.42,2.56) and 1.69 (1.25,2.27). Whilst the respective information for post-predation data was 1.72 (1.35,2.22) and 1.94 (0.98,3.79), as a result, the post-predation mean conditional effect for time period 3 was 19cm lower than non-predation data, whilst post-predation was 25cm higher than the non-predation data for time period 4.

**Table A1.** VOD model summary for non-predation data. Parameter estimates for the model describing the relationship between VOD and the predictor variables. CI, credible interval.

| **Population-Level Effects:** |  |  |  |  |  |  |  |
| --- | --- | --- | --- | --- | --- | --- | --- |
|  | **Estimate** | **Est.Error** | **l-95% CI** | **u-95% CI** | **Rhat** | **Bulk_ESS** | **Tail_ESS** |
| Intercept | 1.06 | 0.11 | 0.83 | 1.28 | 1.00 | 17425 | 16706 |
| Visual orientation distance delay (VODD) | -0.05 | 0.04 | -0.13 | 0.03 | 1.00 | 14112 | 14728 |
| Compatibility (Looking) | 0.04 | 0.05 | -0.06 | 0.14 | 1.00 | 31632 | 19128 |
| Compatibility (Not looking not engaged) | 0.01 | 0.08 | -0.15 | 0.16 | 1.00 | 25503 | 18582 |
| Habitat (Open) | 0.27 | 0.05 | 0.17 | 0.36 | 1.00 | 26782 | 18678 |
| Number of neighbours in 5m | -0.02 | 0.02 | -0.05 | 0.02 | 1.00 | 32494 | 18920 |
| Time period (2) | -0.01 | 0.06 | -0.13 | 0.11 | 1.00 | 26318 | 19642 |
| Time period (3) | 0.09 | 0.06 | -0.03 | 0.21 | 1.00 | 28963 | 19481 |
| Time period (4) | 0.01 | 0.06 | -0.11 | 0.14 | 1.00 | 22580 | 18159 |
| Trial number | 0 | 0.01 | -0.01 | 0.02 | 1.00 | 25632 | 19227 |
|  |  |  |  |  |  |  |  |
| Family Specific Parameters: |  |  |  |  |  |  |  |
| sigma | 0.28 | 0.02 | 0.25 | 0.31 | 1.00 | 17237 | 16861 |
|  |  |  |  |  |  |  |  |
| Group-Level Effects: |  |  |  |  |  |  |  |
| Individual identity (16 levels) |  |  |  |  |  |  |  |
| sd(Intercept) | 0.32 | 0.09 | 0.18 | 0.52 | 1.00 | 12070 | 16101 |
| sd(VODD) | 0.12 | 0.04 | 0.05 | 0.22 | 1.00 | 6637 | 8884 |
| sd(TrialNo) | 0.01 | 0.01 | 0 | 0.02 | 1.00 | 12782 | 13823 |
| cor(Intercept,VODD) | -0.15 | 0.33 | -0.7 | 0.58 | 1.00 | 8601 | 11062 |
| cor(Intercept,TrialNo) | -0.01 | 0.48 | -0.86 | 0.87 | 1.00 | 29082 | 17931 |
| cor(VODD,TrialNo) | -0.04 | 0.49 | -0.88 | 0.85 | 1.00 | 24454 | 18840 |

| **Population-Level Effects:** |  |  |  |  |  |  |  |
| --- | --- | --- | --- | --- | --- | --- | --- |
|  | **Estimate** | **Est.Error** | **l-95% CI** | **u-95% CI** | **Rhat** | **Bulk_ESS** | **Tail_ESS** |
| Intercept | 1.48 | 0.19 | 1.1 | 1.86 | 1.00 | 15054 | 17112 |
| Visual Orientation Distance Delay (VODD) | -0.11 | 0.11 | -0.33 | 0.09 | 1.00 | 11627 | 13721 |
| Compatibility (Looking) | 0.17 | 0.16 | -0.13 | 0.48 | 1.00 | 15486 | 17269 |
| Compatibility (Not engaged not looking) | 0.05 | 0.18 | -0.31 | 0.41 | 1.00 | 19931 | 18047 |
| Habitat (Open) | -0.06 | 0.15 | -0.35 | 0.24 | 1.00 | 16406 | 17308 |
| Number of neighbours within 5m | -0.07 | 0.04 | -0.15 | 0.02 | 1.00 | 17237 | 17283 |
| Time period 4 | 0 | 0.21 | -0.42 | 0.42 | 1.00 | 11925 | 14527 |
| Trial Number 2 post predation | -0.09 | 0.14 | -0.37 | 0.2 | 1.00 | 12759 | 16386 |
| Trial Number 3 post predation | -0.21 | 0.26 | -0.71 | 0.29 | 1.00 | 12330 | 14631 |
|  |  |  |  |  |  |  |  |
|  |  |  |  |  |  |  |  |
| Family Specific Parameters: |  |  |  |  |  |  |  |
| shape | 21.04 | 21.1 | 7.26 | 72.98 | 1.00 | 1468 | 1152 |
|  |  |  |  |  |  |  |  |
| Group-Level Effects: |  |  |  |  |  |  |  |
| Individual identity (16 levels) |  |  |  |  |  |  |  |
| sd(Intercept) | 0.28 | 0.16 | 0.02 | 0.64 | 1.00 | 3490 | 8283 |
| sd(VODD) | 0.18 | 0.1 | 0.02 | 0.39 | 1.00 | 3861 | 6953 |
| sd(TrialNoTrial2) | 0.18 | 0.13 | 0.01 | 0.49 | 1.00 | 3453 | 6528 |
| sd(TrialNoTrial3) | 0.21 | 0.14 | 0.01 | 0.52 | 1.00 | 3214 | 7440 |
| cor(Intercept,VODD) | -0.09 | 0.44 | -0.82 | 0.77 | 1.00 | 7999 | 14009 |
| cor(Intercept,TrialNoTrial2) | -0.26 | 0.44 | -0.9 | 0.69 | 1.00 | 8544 | 16568 |
| cor(VODD,TrialNoTrial2) | -0.03 | 0.43 | -0.81 | 0.78 | 1.00 | 15736 | 17351 |
| cor(Intercept,TrialNoTrial3) | -0.11 | 0.43 | -0.83 | 0.74 | 1.00 | 11569 | 14760 |
| cor(VODD,TrialNoTrial3) | -0.24 | 0.42 | -0.89 | 0.67 | 1.00 | 11981 | 16686 |
| cor(TrialNoTrial2,TrialNoTrial3) | 0.11 | 0.45 | -0.77 | 0.85 | 1.00 | 11261 | 18084 |

**Table A2.** VOD model summary for post-predation data. Parameter estimates for the model describing the relationship between VOD and the predictor variables. CI, credible interval.

**Table A3.** FID model summary for non-predation data. Parameter estimates for the model describing the relationship between FID and the predictor variables. CI, credible interval.

| **Population-Level Effects:** |  |  |  |  |  |  |  |
| --- | --- | --- | --- | --- | --- | --- | --- |
|  | **Estimate** | **Est.Error** | **l-95% CI** | **u-95% CI** | **Rhat** | **Bulk_ESS** | **Tail_ESS** |
| Intercept | 0.66 | 0.16 | 0.35 | 0.97 | 1.00 | 6975 | 12546 |
| Visual orientation distance index (VODI) | -0.13 | 0.05 | -0.23 | -0.03 | 1.00 | 15903 | 15256 |
| Engaged (Not engaged) | 0.07 | 0.06 | -0.04 | 0.18 | 1.00 | 31451 | 18530 |
| Habitat (Open) | 0.22 | 0.06 | 0.11 | 0.33 | 1.00 | 30976 | 19003 |
| Number of neighbours in 5m | -0.06 | 0.02 | -0.1 | -0.01 | 1.00 | 24852 | 18340 |
| Time period (2) | 0.01 | 0.08 | -0.14 | 0.16 | 1.00 | 19478 | 18357 |
| Time period (3) | 0.13 | 0.08 | -0.03 | 0.27 | 1.00 | 20021 | 18781 |
| Time period (4) | 0.01 | 0.08 | -0.14 | 0.16 | 1.00 | 20437 | 18687 |
| Trial number | 0 | 0.01 | -0.01 | 0.02 | 1.00 | 22816 | 17929 |
|  |  |  |  |  |  |  |  |
|  |  |  |  |  |  |  |  |
| Family Specific Parameters: |  |  |  |  |  |  |  |
| sigma | 0.35 | 0.02 | 0.31 | 0.39 | 1.00 | 20819 | 17377 |
|  |  |  |  |  |  |  |  |
| Group-Level Effects: |  |  |  |  |  |  |  |
| Individual identity (16 levels) |  |  |  |  |  |  |  |
| sd(Intercept) | 0.5 | 0.12 | 0.3 | 0.79 | 1.00 | 8819 | 11872 |
| sd(VODI) | 0.11 | 0.06 | 0.01 | 0.25 | 1.00 | 5708 | 6980 |
| sd(TrialNo) | 0.01 | 0.01 | 0 | 0.03 | 1.00 | 9067 | 10206 |
| cor(Intercept,VODI) | 0.22 | 0.39 | -0.55 | 0.89 | 1.00 | 14389 | 12983 |
| cor(Intercept,TrialNo) | -0.28 | 0.46 | -0.93 | 0.73 | 1.00 | 20596 | 16315 |
| cor(VODI,TrialNo) | -0.23 | 0.48 | -0.93 | 0.78 | 1.00 | 14801 | 18491 |

**Table A4.** FID model summary for post-predation data. Parameter estimates for the model describing the relationship between FID and the predictor variables. CI, credible interval.

| **Population-Level Effects:** |  |  |  |  |  |  |  |
| --- | --- | --- | --- | --- | --- | --- | --- |
|  | **Estimate** | **Est.Error** | **l-95% CI** | **u-95% CI** | **Rhat** | **Bulk_ESS** | **Tail_ESS** |
| Intercept | 0.33 | 0.21 | -0.07 | 0.75 | 1.00 | 13782 | 17143 |
| Visual Orientation Distance Index (VODI) | 0.23 | 0.1 | 0.03 | 0.43 | 1.00 | 13948 | 15853 |
| Engaged (Not engaged) | 0.29 | 0.16 | -0.01 | 0.6 | 1.00 | 11678 | 15621 |
| Habitat (Open) | -0.05 | 0.15 | -0.33 | 0.25 | 1.00 | 15930 | 17451 |
| Number of neighbours within 5m | -0.09 | 0.05 | -0.18 | 0 | 1.00 | 11852 | 15558 |
| Time period 4 | 0.12 | 0.32 | -0.49 | 0.76 | 1.00 | 8192 | 10022 |
| Trial Number 2 post predation | -0.05 | 0.19 | -0.44 | 0.31 | 1.00 | 10779 | 10083 |
| Trial Number 3 post predation | -0.31 | 0.34 | -0.99 | 0.36 | 1.00 | 9091 | 10542 |
|  |  |  |  |  |  |  |  |
| Family Specific Parameters: |  |  |  |  |  |  |  |
| shape | 14.03 | 14.54 | 5.61 | 42.82 | 1.00 | 2061 | 1502 |
|  |  |  |  |  |  |  |  |
| Group-Level Effects: |  |  |  |  |  |  |  |
| Individual identity (16 levels) |  |  |  |  |  |  |  |
| sd(Intercept) | 0.24 | 0.15 | 0.02 | 0.57 | 1.00 | 4313 | 8130 |
| sd(VODI) | 0.09 | 0.08 | 0 | 0.29 | 1.00 | 6188 | 8417 |
| sd(TrialNoTrial2) | 0.36 | 0.22 | 0.02 | 0.83 | 1.00 | 2877 | 6859 |
| sd(TrialNoTrial3) | 0.17 | 0.13 | 0.01 | 0.47 | 1.00 | 4606 | 6321 |
| cor(Intercept,VODI) | -0.15 | 0.46 | -0.88 | 0.76 | 1.00 | 19464 | 16743 |
| cor(Intercept,TrialNoTrial2) | -0.06 | 0.42 | -0.79 | 0.77 | 1.00 | 9945 | 14897 |
| cor(VODI,TrialNoTrial2) | -0.02 | 0.43 | -0.8 | 0.79 | 1.00 | 9339 | 14873 |
| cor(Intercept,TrialNoTrial3) | -0.08 | 0.44 | -0.83 | 0.78 | 1.00 | 20489 | 17746 |
| cor(VODI,TrialNoTrial3) | -0.06 | 0.45 | -0.84 | 0.78 | 1.00 | 16192 | 16930 |
| cor(TrialNoTrial2,TrialNoTrial3) | 0.12 | 0.44 | -0.75 | 0.85 | 1.00 | 15488 | 19574 |
